# Metagenomic analyses of a microbial assemblage in a subglacial lake beneath the Vatnajökull ice cap, Iceland

**DOI:** 10.3389/fmicb.2023.1122184

**Published:** 2023-03-30

**Authors:** Pauline Vannier, Gregory K. Farrant, Alexandra Klonowski, Eric Gaidos, Thorsteinn Thorsteinsson, Viggó þór Marteinsson

**Affiliations:** ^1^MATIS, Department of Research and Innovation, Reykjavík, Iceland; ^2^Department of Earth Sciences, University of Hawai’i at Mānoa, Honolulu, HI, United States; ^3^Icelandic Meteorological Office, Reykjavík, Iceland; ^4^Faculty of Food Science and Nutrition, University of Iceland, Reykjavík, Iceland

**Keywords:** subglacial lakes, Iceland, microbial assemblage, metagenome, metabolism

## Abstract

Skaftárkatlar are two subglacial lakes located beneath the Vatnajökull ice cap in Iceland associated with geothermal and volcanic activity. Previous studies of these lakes with ribosomal gene (16S rDNA) tag sequencing revealed a limited diversity of bacteria adapted to cold, dark, and nutrient-poor waters. In this study, we present analyses of metagenomes from the lake which give new insights into its microbial ecology. Analyses of the 16S rDNA genes in the metagenomes confirmed the existence of a low-diversity core microbial assemblage in the lake and insights into the potential metabolisms of the dominant members. Seven taxonomic genera, *Sulfuricurvum*, *Sulfurospirillum*, *Acetobacterium*, *Pelobacter/Geobacter*, *Saccharibacteria, Caldisericum*, and an unclassified member of Prolixibacteraceae, comprised more than 98% of the rDNA reads in the library. Functional characterisation of the lake metagenomes revealed complete metabolic pathways for sulphur cycling, nitrogen metabolism, carbon fixation *via* the reverse Krebs cycle, and acetogenesis. These results show that chemolithoautotrophy constitutes the main metabolism in this subglacial ecosystem. This assemblage and its metabolisms are not reflected in enrichment cultures, demonstrating the importance of *in situ* investigations of this environment.

## 1. Introduction

Subglacial lakes can form where water collects at hydrostatic stable points beneath an ice sheet, when geothermal heat, pressure-induced freezing point depression, and/or high salinity prevent complete freezing (Livingstone et al., 2022). These lakes are relatively isolated ecosystems that can host life despite low temperatures, low nutrient abundance, and the absence of sunlight as an energy source. Terrestrial subglacial lakes are considered accessible if imperfect, analogues to ice-covered environments in the past and present Solar system: the ice-covered oceans of “Snowball” Earth (Kirschvink et al., 2000), lakes beneath the south polar cap of Mars (Lauro et al., 2020) and oceans in some of the icy satellites of Jupiter and Saturn (Kivelson et al., 2000, 2002; Thomas et al., 2016). Studies of these systems complement research on other lakes with comparatively thin (metres) ice covers, e.g., the Dry Valleys lakes (Chinn, 1993) and epishelf lakes of Antarctica (Davies et al., 2017), where sufficient photosynthetic active radiation can reach the water column and support phototrophic communities (Howard-Williams et al., 1998).

There are more than 700 reported subglacial lakes in Antarctica (Livingstone et al., 2022), three in Iceland under the Vatnajökull glacier (Bjornsson, 2003), two in Greenland (Palmer et al., 2013), and one recently discovered in the Canadian Arctic (Rutishauser et al., 2018). The biological exploration of some of these lakes has begun, namely Lake Vostok (Karl et al., 1999; Priscu et al., 1999; Christner et al., 2001; Bulat, 2016; Gura and Rogers, 2020), Subglacial Lake Whillans (Tulaczyk et al., 2014), Subglacial Lake Mercer in Antarctica (Priscu et al., 2021), and all three Icelandic lakes (Gaidos et al., 2004, 2009; Marteinsson et al., 2013). In the cases of Antarctica and Iceland, the data unambiguously point to the presence of active assemblages of bacterial taxa which are distinct from the distribution in the overlying ice or surrounding glaciated terrain (Gaidos et al., 2004, 2009; Marteinsson et al., 2013; Achberger et al., 2016).

The three main Icelandic lakes, called Grímsvötn, Western, and Eastern Skaftárkatlar lakes, are distinguished by their location on active volcanoes and are maintained by geothermal melting at the base of the 250–300-m thick Vatnajökull ice cap. The chemistry of the approximately 100-m-deep water columns of these lakes is substantially influenced by volcanic gases and hydrothermal fluids, maintaining an anoxic and highly sulphidic environment (Agustsdottir and Brantley, 1994; Johannesson et al., 2007). Mixing of anoxic lake water with oxygenated glacial meltwater creates chemical disequilibrium that can serve as an energy source for chemolithotrophic microorganisms (Gaidos et al., 2004, 2009; Marteinsson et al., 2013). Previous investigations have used molecular tag-based methods to identify the dominant microbial taxa in the water column of the Skaftárkatlar lakes and link chemical reactions of the inorganic substrates thought present in the lakes with potential metabolisms of these taxa, e.g., acetogenesis, sulphide oxidation, sulphate reduction, iron reduction, and hydrogen oxidation by members of *Acetobacterium*, *Geobacter*, *Sulfuricurvum*, *Sulfurospirillum*, and *Desulfosporosinus* (Gaidos et al., 2009; Marteinsson et al., 2013). In Subglacial Lake Whillans, the chemoautotrophic microbial taxa, distinct from that taxa of the Icelandic subglacial lakes, use reduced nitrogen, iron or sulphur compounds as energy sources (Christner et al., 2014).

Here, we performed a metagenomic analysis on four water column samples collected from the Eastern Skaftárkatlar lake in 2007, as well as on enrichment cultures from the same samples (Marteinsson et al., 2013). Our study aimed to: (1) confirm the previously reported microbial community structure, (2) investigate the metabolic strategies of the microorganisms in the lake, and (3) analyse potential pathways within microbes grown in enrichment cultures under different conditions. Compared to the previous study done on the same water samples but only with a 16S rRNA sequencing approach, we adopted a metagenomic approach as a more informative, less biassed method to gain further understanding of the microbial diversity and community structure but also and most importantly of the functional capabilities both at the taxon and community level occurring in such a specific environment. Our analyses show unambiguously that the microbial communities of this Icelandic subglacial lake are dominated by seven taxonomic genera: *Sulfuricurvum*, *Sulfurospirillum*, *Acetobacterium*, *Pelobacter/Geobacter*, *Saccharibacteria, Caldisericum*, and an unclassified Prolixibacteraceae. Chemolithoautotrophy is the main metabolism of these communities and is adapted to their environment with oxidation/reduction of sulphur, nitrogen metabolism, carbon fixation *via* the reverse Krebs cycle, and acetogenesis.

## 2. Materials and methods

### 2.1. Environmental samples

Water column samples were collected from the East Skaftárkatlar lake, in June 2007 as described by Marteinsson et al. (2013). Two boreholes A and B were drilled through the overlying 280-m-thick ice sheet with a sterilising hot water drill (Thorsteinsson et al., 2008). Each collected sample was about 1 L and the results of chemical analyses of the samples (Marteinsson et al., 2013) are reported in Supplementary Table 1. As B_1_ and B_4_ samples appeared chemically homogeneous (Supplementary Table 1), a pool of the B_1_ to B_4_ samples was created, B_mix_, to increase total DNA yield.

### 2.2. Enrichment samples

The E_mix_ sample consists of biomass pooled from enrichment cultures that showed growth as previously described (Marteinsson et al., 2013). Media were designed to cultivate chemolithotrophs and chemo-organotrophs at 4, 60, and 80°C. Briefly, different enrichment media were used and inoculated with 1% of water lake samples under anaerobic conditions. Sample of 0.22 μm micron-filtered lake water was used as a medium for enrichment cultures labelled “WN_2_,” “WO_2_,” “C-J,” and “A-J” for respectively “Water N_2_,” “Water O_2_,” “Enrichment C-jökull” (meaning glacier in Icelandic), and “Enrichment A-jökull.” A supplement yeast extract solution (0.01%), vitamin solution, Balch element solution (Balch et al., 1979), S^0^, and resazurin were added to WN_2_ and WO_2_ enrichment cultures. WO_2_ was used aerobically whereas WN_2_ was incubated with pure N_2_ and supplemented with 0.025% final wt v^–1^ Na_2_S.9H_2_O. A volume of sterile water was supplemented with 1X of yeast-acetate medium (Gaidos et al., 2009) and used as media for C-J and A-J. C-J was incubated with 80/20% H_2_/CO_2_ and 0.025% final wt. v^–1^ Na_2_S.9H_2_O whereas A-J was incubated aerobically. Finally, enrichment cultures were done aerobically with 162 Thermus medium (Degryse et al., 1978) and Reasoner’s 2A (R_2_A) medium (Reasoner and Geldreich, 1985) whereas Thermotoga and Yeast Peptone Sulphur (YPS) media were used anaerobically with pure N_2_ (Marteinsson et al., 1997, 2001). Pellets of cells from these enrichments were obtained by centrifugation at 8,000 rpm for 25 min and used for DNA extraction.

### 2.3. DNA extraction and sequencing

DNA was extracted from filtered water samples and enrichment cultures as previously described (Gaidos et al., 2009). Three different DNA samples were sequenced: A_3_, B_mix_, and E_mix_ (Table 1). The DNA was sent to the Marine Biological Laboratory at the Woods Hole Institute for sequencing on an Illumina HiSeq (Illumina, Inc., CA, USA) as part of the Census of Deep Life of the Deep Carbon Observatory. DNA was sheared using a Covaris S2 sonicator (Covaris, Woburn, MA, USA) and libraries were constructed with the Nugen Ovation Ultralow Library protocol (NuGEN Technologies, San Carlos, CA, USA). Expected insert size of 175 bp enabled overlapping reads. Amplified libraries were visualised on an Agilent DNA1000 chip (Agilent, Santa Clara, CA, USA) or Caliper HiSens Bioanalyzer assay (Perkin Elmer, Waltham MA, USA), pooled at equimolar concentrations and size selected using a Sage PippinPrep 2% cassette (Sage Science, Beverly, MA, USA). The library pool was quantified using a Kapa Biosystems qPCR library quantification kit (Kapa Biosystems, Wilmington, MA, USA), then sequenced on the HiSeq1000 (Illumina) in a 2 × 108 bp paired-end sequencing run using dedicated read indexing. The samples were then demultiplexed (barcode-based sorting of sequences from different samples) with CASAVA (v.1.8.2; Illumina) while removing the Illumina adaptors. Reads were then merged using FLASH v1.2.11 (Magoč and Salzberg, 2011) with default parameters. Short reads and low-quality bases were then removed using the clc_quality_trim command in CLC (v4.4.0.122465; options used: minlength 90, badfraction 0, i.e., no low-quality nucleotides allowed; CLC Bio, Aarhus, Denmark).

**TABLE 1:** Sample information from East Skaftárkatlar lake.

Sample name	Sample type	Depth (metres)	DNA concentration (ng/μl)
**A_3_**	Water	379	56
B_1_	Water	284	15
B_2_	Water	336	31
B_3_	Water	377	22
B_4_	Water	388	68
**B_**mix**_**	DNA pool of B_1_, B_2_, B_3,_ and B_4_	–	136
E_3_	Enrichment culture at 4°C	–	246
E_60_	Enrichment culture at 60°C	–	269
E_80_	Enrichment culture at 80°C	–	158
**E_**mix**_**	DNA pool of E_3_, E_60,_ and E_80_	–	673

The bold font was to highlight the samples that were analyzed in this article.

### 2.4. Biodiversity analysis based on rDNA _mi_TAGs

Reads corresponding to rDNA genes, a.k.a. _mi_TAGs for metagenome Illumina tags (Logares et al., 2014), were extracted from the metagenomes using Hidden Markov Models (HMM) in Meta-RNA (Huang et al., 2009). The reads were assembled on Geneious (R10, Biomatters, Auckland, New Zealand) with conservative settings (“Fastest,” <1% mismatch, otherwise default parameters) to avoid chimeric assembly. Using iterative mappings of the rest of the reads with Geneious (Biomatters), the contigs were manually extended and assembled-when the coverage was sufficient- into complete ribosomal operons containing the 16S, 23S, and 5S rDNA genes and homogeneous coverage, allowing more reliable taxonomic assignments than with partial or single genes. These assembled rDNA genes were then annotated using either BLASTN+ against public databases NT and SILVA (Camacho et al., 2009; Quast et al., 2012) or the online RDP classifier (Wang et al., 2007). Most contigs get a significant assignment (>98% 16S identity) with the notable exception among the dominant taxa of *Caldisericum* sp. (96% identity against the closest 16S barcode and only 83% identity against the closest genome of *Caldisericum exile*). The rDNA genes were then aligned and trimmed to about 1,819; 3,731, and 115 bp (for 16S, 23S, and 5S, respectively) and used as references to recruit the ribosomal fraction of the raw metagenomic reads with high stringency (≥50% alignment coverage and ≥98% identity). From this point, we assumed that each assemblage represents a distinct taxon. To better reflect the actual community structure, read counts per taxon were then normalised based on actual gene length and the number of copies of the operon in each taxon. The estimation of the copy number of each ribosomal operon was obtained from ribosomal RNA operon copy number database (rrnDB) (Stoddard et al., 2014) by rounding the average number of rDNA operon copies at the taxonomic level retained for each operon (see Table 2).

**TABLE 2:** Genetic materials of East Skaftárkatlar lake and associated analyses used in this study (*A_3_-B_mix_ co-assembly).

	Sample name	A_3_	B_mix_		E_mix_
*De novo* Illumina sequencing, merging, and cleaning	Amount of DNA (ng)	56	136		673
	Number of pair-end reads (x2)	35,404,237	30,780,977		32,362,167
	Number of merged base-pairs	5,262,627,789	4,552,871,144		4,320,910,461
	Average length (bp)	157.05	155.85		151.59
rDNA read extraction and assembly	Number of rDNA reads	209,089	175,427		80,189
	Number of rDNA sequences	16S: 27 – 23S: 26 – 5S: 26			
		**A_3_**	**B_mix_**	A_3_-B_mix_*	**E_mix_**
2-steps metagenome assembly	Number of contigs	61,052	47,708	64,905	44,111
	Number of base pairs	60,396,455	68,986,232	83,643,503	66,501,590
	Minimum length (bp)	80	78	80	79
	Maximum length (bp)	247,938	357,766	279,868	1,472,397
	Average length (bp)	989.26	1,446.01	1,288.71	1,507.60
	N50	2,502	6,373	5,182	5,533
	N90	348	413	377	424
ORFs detection	Number of ORFs	95,010	99,688	124,531	97,036
	Minimum length (bp)	57	57	57	57
	Maximum length (bp)	10,023	12,087	12,756	11,385
	Average length (bp)	526.29	594.54	570.19	608.75

Additionally, binning experiments based on nucleotide composition, GC%, and differential coverage in A_3_, B_mix_, and E_mix_ [CONCOCT (Alneberg et al., 2014)] were tried and failed at properly separating some closely related taxa, partly due to an overall low sequencing depth and lack of variation in the taxonomic profile of available samples.

### 2.5. Microbial metagenome, functional potential

**FIGURE 1:**
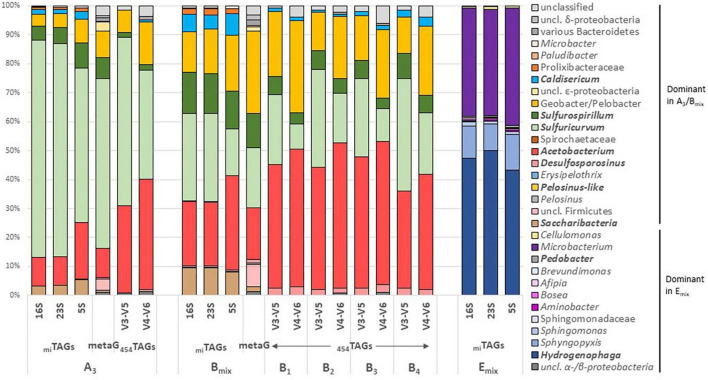
Community structure of metagenomes A_3_, B_mix_, and E_mix_ from East Skaftárkatlar lake based on metagenome extracted rDNA _mi_TAGs. Assignments were performed using BLASTN against SILVA 128 (Quast et al., 2012; Yilmaz et al., 2013).

With regards to their comparable community composition shown through the rDNA _mi_TAG analyses (see section “Results” and Figure 1), the raw metagenomes A_3_ and B_mix_ were co-assembled *de novo* to improve the functional analyses. At first, default configurations of IDBA-UD (Peng et al., 2012) and SPAdes (Bankevich et al., 2012) were run to generate reliable contigs. The resulting contigs were then assembled into “supercontigs” using Geneious (Biomatters; default “Fastest” with 2% mismatch allowed). These “supercontigs” and the other unassembled original contigs were used for the functional exploration of the water column metagenome. MetaGeneMark (Zhu et al., 2010) was used online with default parameters to detect open reading frames (ORFs) even truncated at the edge of contigs. Function and taxonomy were then assigned to these genes by amino-acid alignment against the Kyoto Encyclopedia of Genes and Genomes database (KEGG, FTP Release 12-02-2018) (Kanehisa and Goto, 2000; Kanehisa et al., 2015) using DIAMOND (with 60% identity cut-off) (Buchfink et al., 2015) and their abundance in the metagenome was estimated by recruiting the raw metagenomics reads using BLASTN (Camacho et al., 2009). The results were manually checked and when 80% of the genes involved in a pathway were detected, the pathways were considered as present. Errors in the taxonomic assignments of the ORFs are generally caused by low taxonomic resolution or the absence of close relatives in the reference dataset and may result on genes being assigned to close relatives of the represented diversity. These were reduced by collapsing branches of the trees using TaxonomyCollapsor.^1^ This algorithm sorts the leaves on the phylogenetic trees according to decreasing abundance (as read counts) and flags as significant those for which the cumulated abundance contains 95% of the total abundance. Leaves that do not reach the significance criteria are then reassigned by order of priority to their closest significant relative of the same rank or at a higher taxonomical level.

## 3. Results

### 3.1. Metagenome sequence yield

DNA was extracted and sequenced from samples collected from the bottom of the lake (sample A_3_), from a mixture of four water column samples collected at different depths (sample B_mix_), and from enrichments grown under various culture conditions (sample E_mix_) (Tables 1, 2). The metagenomes A_3_ and B_mix_ allow us to investigate the microbial community of the lake water column whereas E_mix_ permits to identify the growing organisms that might be rare in the environment but can be enriched under laboratory conditions. The sequencing of all the DNA available for A_3_, B_mix_, and E_mix_ resulted in 30–35 × 10^6^ overlapping paired reads per sample with respectively an average merged length of 157.05 bp (σ = 15.65 bp), 155.85 bp (σ = 19.84 bp), and 151.59 bp (σ = 18.96 bp) (Table 2).

### 3.2. Diversity and community structure based on ribosomal DNA sequences

Hidden Markov Models were used to identify 209,089, 175,427, and 80,189 rDNA reads in the A_3_, B_mix_, and E_mix_ libraries, respectively (Table 2). Conservative *de novo* assembly and manual improvements of these assemblies yielded 25 distinct high-quality full-length rDNA operons (each consisting of 5S, 16S, and 23S genes and intergenic regions) and 4 partial operons (missing all or part of a rDNA gene) (Table 3 and Supplementary Table 2). The libraries sequenced from the natural samples A_3_ and B_mix_ both contain seven taxa with relative read abundance >1%, namely *Acetobacterium* sp., *Sulfuricurvum* sp., *Sulfurospirillum* sp., *Geobacter* sp./*Pelobacter* sp., *Caldisericum* sp., *Saccharibacteria* sp., and an unclassified Prolixibacteraceae. Together, those taxa recruit 98.0 to 99.2% of the total rDNA reads in A_3_ and B_mix_, respectively. Based on 16S-rDNA _mi_TAGs, Shannon’s α-diversity is 1.18 and 1.83 for A_3_ and B_mix_, respectively, while evenness is 0.14 and 0.33, respectively. While the A_3_ metagenome is dominated by *Sulfuricurvum* sp. (74% of the 16S reads), followed by *Acetobacterium* (16%), B_mix_ seems to be more evenly distributed, consisting of *Sulfuricurvum* (30%), *Acetobacterium* sp. (22%), *Sulfurospirillum* (14%) and *Geobacter* sp./*Pelobacter* sp. (13%). The community structure observed for the three samples based on 16S-, 23S-, and 5S-rDNA _mi_TAGs [metagenome Illumina tags (Logares et al., 2014)] was compared to that observed in samples A_3_ and B_1–4_ obtained by Marteinsson et al. (2013) using 16S rDNA pyrosequencing (Figure 1).

**TABLE 3:** rDNA contig assembly results.

Complete taxonomy	Assignment	Copy number	16S identity	23S identity	5S identity	Origin
Bacteria; Epsilonproteobacteria; Campylobacterales; Helicobacteraceae*; Sulfuricurvum kujiense*	*Sulfuricurvum kujiense*	3	99%	97%	99%	N
Bacteria; Firmicutes; Clostridia; Clostridiales; Eubacteriaceae; *Acetobacterium woodii*	*Acetobacterium woodii*	5	n.d.	96%	99%	N
Bacteria; Deltaproteobacteria; Desulfuromonadales; Desulfuromonadaceae; *Geobacter* Bacteria; Deltaproteobacteria; Desulfuromonadales; Desulfuromonadaceae; *Pelobacter*	*Geobacter*/*Pelobacter* sp.	4	98%	96%	99%	N
Bacteria; Epsilonproteobacteria; Campylobacterales; Campylobacteraceae; *Sulfurospirillum*	*Sulfurospirillum* sp.	2	98%	98%	94%	N
Bacteria; Epsilonproteobacteria; Campylobacterales; Campylobacteraceae; *Sulfurospirillum*	*Sulfurospirillum* sp. TAX2	2	n.d.	97%	95%	N
Bacteria; unclassified Bacteria; Bacteria candidate phyla; Candidatus Saccharibacteria	Unclassified Saccharibacteria	1	99% (94%)	91%	96%	N
Bacteria; Caldiserica; Caldisericia; Caldisericales; Caldisericaceae; *Caldisericum*	*Caldisericum* sp.	1	96% (83%)	<78%	*No hit*	N
Bacteria; Bacteroidetes; Bacteroidia; Bacteroidales; Prolixibacteraceae	Unclassified Prolixibacteraceae	2	98% (89%)	85%	94%	N
Bacteria; Bacteroidetes; Bacteroidia; Bacteroidales; Porphyromonadaceae; *Microbacter*	*Microbacter* sp.	3	97% (95%)	88%	92%	N
Bacteria; Bacteroidetes; Bacteroidia; Bacteroidales; Paludibacteraceae; *Paludibacter*	*Paludibacter* sp.	3	99% (96%)	88%	98%	N
Bacteria; Firmicutes; Clostridia; Clostridiales; Peptococcaceae; *Desulfosporosinus*	*Desulfosporosinus* sp.	11	97% +5 var.	91% +2 var.	93%	N
Bacteria; Spirochaetes; Spirochaetia; Spirochaetales; Spirochaetaceae	Unclassified Spirochaetaceae	2	96%	83%	85%	N
Bacteria; Firmicutes; Erysipelotrichia; Erysipelotrichales; Erysipelotrichaceae; *Erysipelothrix*	*Erysipelothrix* sp.	5	99% (91%)	89%	88%	N
Bacteria; Firmicutes; Negativicutes; Selenomonadales; Sporomusaceae; *Pelosinus* sp.	*Pelosinus* sp. TAX1	12	95%	88%	85%	N
Bacteria; Firmicutes; Negativicutes; Selenomonadales; Sporomusaceae; *Pelosinus* sp.	*Pelosinus* sp. TAX2	12	98%	95%	93%	N
Bacteria; Betaproteobacteria; Burkholderiales; Comamonadaceae; *Hydrogenophaga*	*Hydrogenophaga* sp.	1	99%	99%	97%	E
Bacteria; Actinobacteria; Micrococcales; Microbacteriaceae; *Microbacterium*	*Microbacterium* sp.	2	100%	100%	100%	E
Bacteria; Alphaproteobacteria; Sphingomonadales; Sphingomonadaceae; *Sphingopyxis bauzanensis*	*Sphingopyxis bauzanensis*	1	99%	n.d.	n.d.	E
Bacteria; Alphaproteobacteria; Sphingomonadales; Sphingomonadaceae; *Sphingopyxis fribergensis*	*Sphingopyxis fribergensis*	1	99%	99%	100%	E
Bacteria; Alphaproteobacteria; Sphingomonadales; Sphingomonadaceae; *Sphingomonas*	*Sphingomonas* sp.	2	98%	94%	96%	E
Bacteria; Alphaproteobacteria; Sphingomonadales; Sphingomonadaceae	Unclassified Sphingomonadaceae	2	98%*	n.d.	n.d.	E
Bacteria; Actinobacteria; Micrococcales; Cellulomonadaceae; *Cellulomonas*	*Cellulomonas* sp.	2	98%	96%	97%	E
Bacteria; Alphaproteobacteria; Rhizobiales; Phyllobacteriaceae; *Aminobacter*	*Aminobacter* sp.	3	100%	100%	100%	E
Bacteria; Bacteroidetes; Bacteroidia; Sphingobacteriales; Sphingobacteriaceae; *Pedobacter*	*Pedobacter* sp.	1	99%	92%	99%	E
Bacteria; Alphaproteobacteria; Rhizobiales; Bradyrhizobiaceae; *Bosea*	*Bosea* sp.	2	99%	98%	100%	E
Bacteria; Alphaproteobacteria; Rhizobiales; Bradyrhizobiaceae; *Afipia*	*Afipia* sp.	1	96%	98%	100%	E
Bacteria; Alphaproteobacteria; Caulobacterales; Caulobacteraceae; *Brevundimonas*	*Brevundimonas* sp.	2	100%	96%	98%	E
Bacteria; Deltaproteobacteria; Desulfuromonadales; Desulfuromonadaceae; *Geobacter*	*Geobacter* sp.	2	95–97%	94%*	n.d.	E
Bacteria; Firmicutes; Bacilli; Bacillales; Staphylococcaceae; *Staphylococcus pasteuri*	*Staphylococcus pasteuri*	5	100%	99%	100%	C

Assignment and taxonomy are based on SILVA and/or NCBI’s NT. Origin: N, natural sample A_3_/B_mix_; E, E_mix_; C, contamination. Unless stated otherwise, the contigs fully cover the ribosomal gene. *Gene fragment, (xx%) best hit on a genome, when very different, n.d.: not detected. The number of copies of each operon was estimated from the closest relatives at rrndb.umms.med.umich.edu.

Unlike these first four taxa, which are relatively close to their cultivated relatives (over 97% identity), the lower abundance taxa *Caldisericum* sp. candidate (1.7% of 16S-rDNA _mi_TAGs in A_3_, 6.0% in B_mix_) and *Saccharibacteria* sp. (3.3% in A_3_, 9.5% in B_mix_) are more distant from their closest relatives in public databases. The *Caldisericum* sp. candidate 16S and 23S assembled contigs show an alignment identity of 83% with publicly available *C. exile* AZM16c01 (Mori et al., 2009). *Saccharibacteria* sp. rDNA contigs show 94% identity (16S and 23S) with the genome of Candidatus *Saccharibacteria* bacterium GW2011_GWC2_44_17 (Brown et al., 2015), which belongs to the recently described phylum Saccharibacteria (Ferrari et al., 2014). Other taxa were also found belonging to the Prolixibacteraceae family or the *Microbacter* genus and to *Paludibacter*, *Desulfosporosinus*, *Brevundimonas*, and *Pelosinus* in A_3_. Taxa related to members of *Desulfosporosinus*, *Paludibacter*, and *Pelosinus* were also detected in B_mix_ as well as *Erysipelothrix* and *Hydrogenophaga*. In contrast, about 97% of the _mi_TAGs in E_mix_ were recruited by four full-length ribosomal operons assigned to *Hydrogenophaga* sp. (49%), *Microbacterium* sp. (36%), *Sphingopyxis* sp. (10%), and *Sphingomonas* sp. (1.2%) (Figure 1 and Table 3).

A dozen different short (about 100 bp) fragments of 16S rDNA genes that showed identity >95% to various archaeal lineages (*Crenarchaeota*, *Euryarchaeota*, and *Thaumarchaeota*) were assembled from A_3_ and E_mix_. The corresponding reads originally accounted for about 7% of the raw 16S reads extracted from metagenomes A_3_ and E_mix_. The full-length 16S rDNA gene of Euryarchaeota *Candidatus Methanoplasma termitum* MpT1 (CP010070) was used as a reference to recruit the corresponding reads in A_3_ (Supplementary Figure 1). Recruited reads do not homogeneously cover the reference gene, instead, all the reads match two overlapping segments of the reference 16S located between positions 850–957 and 891–1,000. Hence, no full-length 16S rDNA belonging to Archaea was detected in the metagenome and those fragments were considered artefacts and discarded from the rDNA pool used for community structure evaluation.

### 3.3. Metabolic profiling of the subglacial East Skaftárkatlar lake microbiome

**FIGURE 2:**
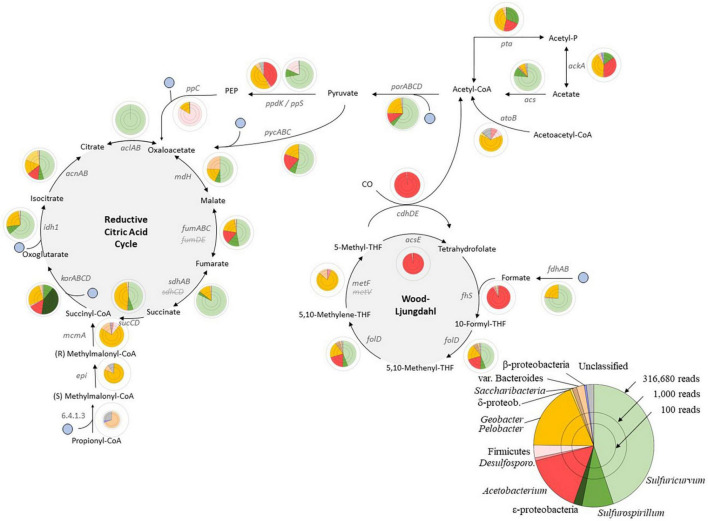
Carbon fixation in East Skaftárkatlar lake and global functional gene recruitment profile. Circular diagrams represent the abundance and taxonomic assignment of the associated reads, with the radius representing the log(nb of reads). 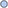 CO_2_/HCO_3_^–^.

The conservative co-assembly of A_3_ and B_mix_ resulted in 64,905 contigs (assembly contiguity statistics: N50 = 5,182 bp, N90 = 377 bp) (Table 2). The online MetaGeneMark algorithm detected 124,531 ORFs in these contigs to which we assigned functions and analysed further. Using a 60% amino acid identity cut-off, we identified 58,203 ORFs (47%) having a significant hit against genes from the KEGG (Kanehisa et al., 2015). Those taxa that dominated the miTAGs analyses (*Acetobacterium*, *Sulfuricurvum*, *Sulfurospirillum*, and *Geobacter/Pelobacter*) also dominated these identified ORFs (Figure 2).

#### 3.3.1. Carbon fixation

Nearly complete pathways for the reductive citric acid cycle and acetogenesis (Wood-Ljungdahl cycle), both allowing fixation of CO_2_ or HCO_3_^–^, were identified (Figure 2). *Sulfuricurvum*, *Sulfurospirillum*, and *Geobacter/Pelobacter* taxa were found to have most of the genes involved in the reductive citric acid cycle. The genes coding for subunits of fumarate hydratase (*fumD* and *fumE*) and succinate dehydrogenase (*sdhC* and *sdhD*) were not detected.

### 3.3.2. Sulphur metabolism

**FIGURE 3:**
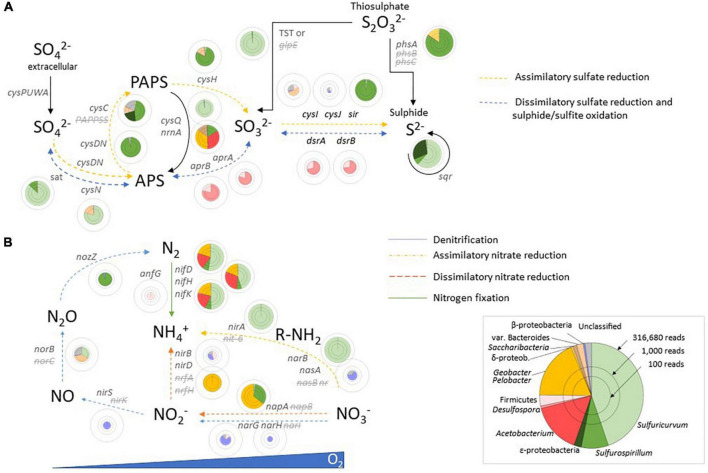
Sulphur metabolism **(A)** and nitrogen metabolism **(B)** in East Skaftárkatlar lake. APS, adenosine-5-phosphosulfate; PAPS, 3-phospho adenosine-5-phosphosulfate; PEP, phosphoenolpyruvate.

Complete pathways for assimilatory sulphate reduction and dissimilatory reduction of sulphur species were detected with notable taxonomic specificity (Figure 3B). The genes coding for sulphate adenylyltransferase (*sat*), adenylylsulphate reductase (*aprAB*), and dissimilatory sulphite reductase (*dsrABC*) were identified and belong mainly to *Desulfosporosinus* and *Acetobacterium*. These enzymes take sulphate to adenosine-5-phosphosulfate (APS), APS to sulphite, and sulphite to sulphide for dissimilatory reduction of sulphate. Thiosulfate might be oxidised to sulphate by *Sulfuricurvum via* the thiosulfate sulfurtransferase (TST).

This sequence of genes does not appear in *Sulfurospirillum* and instead the presence of a polysulphide reductase chain A (*phsA*), or a homologue of *phsA*, suggests that reduction of sulphur species for energy conservation only includes reduction of elemental sulphur or thiosulphate to sulphate in this taxon. Genes coding for assimilatory sulphate reduction such as sulphate adenylyltransferases subunits 1 and 2 (*cysDN*), adenylylsulphate kinase (*cysC*), phosphoadenosine phosphosulphate reductase (*cysH*), and sulphite reductase (ferredoxin) (*sir*) were indeed assigned preferentially to *Sulfurospirillum*.

Moreover, the main enzymes involved in sulphur-disproportionation were not detected in the co-assembly metagenome. In that pathway, thiosulphate, sulphite, or elemental sulphur can serve as both electron or acceptor donors and are converted into sulphate and hydrogen sulphide.

### 3.3.3. Nitrogen metabolism

Some of the genes involved in nitrogen metabolism were detected in the metagenome (Figure 3B). No taxon has a complete assimilatory nitrate reduction pathway. The genes coding for the ferredoxin-nitrate reductase (*narB*) and the catalytic subunit of the assimilatory nitrate reductase (*nasA*) were detected but not the genes coding for the NADPH nitrate reductase (NR) or the assimilatory nitrate reductase electron transfer (*nasB*). Likewise, the gene coding for the ferredoxin-nitrite reductase (*nirA*) was detected but not the gene coding for the assimilatory nitrite reductase (*nit*-6). The detected genes are mainly taxonomically related to *Sulfuricurvum.* Some of the genes involved in dissimilatory nitrate reduction were found: *napA* but not *napB* from the cluster of genes coding the NapAB protein and *narG*, *narH* but not *narI* from the NarGHI cluster. Nitrite can then be reduced to ammonia with the nitrite reductase coded by the *nirB* and *nirD* genes found in our metagenomes. Genes involved in dissimilatory nitrate reduction seem to be related mainly to *Geobacter/Pedobacter*. Nitrogen fixation seems to be widespread in the Skaftárkatlar biome as all the genes coding for the dinitrogenase (*nifD*, *nifK*, *nifH*, and *anfG*), a molybdenum-iron protein reducing dinitrogen to ammonia, were detected, and were assigned to *Sulfurospirillum*, *Sulfuricurvum*, *Acetobacterium*, and *Geobacter/Pelobacter*.

### 3.3.4. Hydrogen

Potential utilisation of dihydrogen was investigated, revealing the presence of genes coding for the quinone-reactive Ni/Fe-hydrogenase small (*hydA*) and large subunit (*hydB*) and assigned to *Sulfuricurvum* and *Sulfurospirillum*. The genes coding for the different NADP-reducing hydrogenase subunits (*hndB, hndC*, and *hndD*) were identified to belong to *Acetobacterium*. The presence of these genes supports the use of H_2_ as an electron donor in these taxa.

### 3.3.5. Other metabolisms

Gene coding for arsenate reductase with disulphide as an acceptor was found (*arsC*) and assigned to *Sulfuricurvum* and *Sulfurospirillum*. Sulphide oxidation can be tied to the reduction of arsenate to arsenide by arsenate respiration. This ability was described for *Sulfurospirillum* (Stolz et al., 1999). Genes involved in anaerobic fumaric respiration such as fumarate reductase, flavoprotein subunit (*frdA*), and iron-sulphur subunit (*fdrB*) were identified and assigned to *Sulfuricurvum*, *Sulfurospirillum*, and *Geobacter/Pelobacter*. Only the *cooS* gene from the gene cluster coding for enzymes allowing anaerobic carboxydotrophy was detected and affiliated to *Acetobacterium*. Genes coding for enzymes involved in the dissimilatory reduction of Fe^3+^ were not detected.

## 4. Discussion

This study presents the first analysis of metagenomes originating from volcanic subglacial lake: one sample collected from the bottom of the lake (A_3_), a second from four pooled water column samples collected from different depths in the lake (B_mix_), and a third from combined enrichments, i.e., growth under conditions that attempted to mimic those in the subglacial lake (E_mix_). The metagenomic analysis of the collected samples enabled a reconstruction of the potential metabolic pathways existing in the lake and a more robust description of the microbial community structure than before. Environmental metagenome analysis such as these have been shown to be more quantitative for microbial community studies, compared to amplicon-tag-based studies, due to the absence of Polymerase Chain Reaction (PCR) amplification bias (Parada et al., 2016; Brumfield et al., 2020).

This study not only confirms a previous study of the lake using 16S rRNA tag sequencing and Fluorescence *In Situ* Hybridisation (FISH) (Marteinsson et al., 2013) but also provides more robust evidence that only a few bacterial taxa dominate the microbial community in the lake water column (*Sulfuricurvum*, *Acetobacterium*, *Geobacter*/*Pelobacter*, and *Sulfurospirillum*). Metagenomic analyses in this study also confirm the presence of lower abundance taxa (e.g., *Caldisericum* and *Desulfosporosinus*) and reveal new additional lineages (*Saccharibacteria* and *Pelosinus*) which had not been observed by the previous amplicon-based studies. The relatively low microbial diversity (∼20 taxa) represented in the metagenome of this ecosystem resulted in high sampling depth and allowed assemblage of full-length 16S, 23S, and often also 5S sequences for most of the taxa. Full-length sequences permitted a more precise taxonomic assignment of the most abundant community members as well as 14 minor taxa with relative abundance ranging from 0.05 to 1%. Many of these taxa are closely related to psychrotolerant strains, including a member of *Brevundimonas*, a taxon detected in the Arctic (Trivedi et al., 2018) and Antarctic (González-Toril et al., 2009) and which is known to be resistant to cold temperatures (Dartnell et al., 2010) and the genus *Pedobacter*, also found in Arctic environments (Zhou et al., 2012; Peter and Sommaruga, 2016; Trivedi et al., 2018).

In a broader context, this study has confirmed previous results (Marteinsson et al., 2013) that the community in the East Skaftárkatlar lake is substantially different from that of other subglacial lakes such as Subglacial Lake Whillans in Antarctica. The former is dominated by Betaproteobacteria such as *Polaromonas*, *Sideroxydans* or *Thiobacillus*, Bacteroidetes, and Actinobacteria (Achberger et al., 2016) while the East Skaftárkatlar lake is dominated by Epsilonbacteria: *Sulfuricurvum* and *Sulfurospirillum*, and Firmicutes: *Acetobacterium*. East Skaftárkatlar could be significantly influenced by hydrothermal activity emanating from the underlying lake bed and thus can host, among others, sulphur oxidisers or reducers (Johannesson et al., 2007). Furthermore, the lake is a mix of glacial melt (containing oxygen) as well as sulphide from disproportionation of dissolved volcanic SO_2_ that supports a sulphur cycle. Then, *Sulfurospirillum deleyianum* is known to be able to oxidise sulphide with nitrate, producing ammonium and intracellular elemental sulphur (Eisenmann et al., 1995). This ability is also known in *Sulfuricurvum* species, the most abundant member of the microbial community in the sample collected at the bottom of the lake 75% (A_3_) compared to 30% in the water column samples (B_mix_). The relative abundance of *Sulfuricurvum* correlates with the sulphate concentration, which was about five times higher in A_3_ than in the other pooled sample B_mix_ (4.71 ppm in A_3_ *vs.* from 0.29 to 1.43 ppm in B_1_ to B_4_, Supplementary Table 1). Compared to A_3_, a higher diversity was observed in B_mix_ (Shannon’s α-diversity of 1.18 and 1.83, respectively, data not shown) including three additional taxa: Peptococcaceae, Bacteroidetes, and *Caldisericum* spp. which might indicate that growth and metabolism are faster than the mixing time of the water column across any vertical or lateral chemical gradients in the lake. The higher diversity could also be a result of the fact that B_mix_ is derived from a mixture of samples.

Despite many different enrichment conditions, none of the dominant taxa found in the water column samples were enriched under the conditions of our incubations. The absence of culturable members of the main diversity is not unexpected as uncultivated phyla frequently dominate diverse environments (Lloyd et al., 2018). Interestingly, the dominant taxa found in E_mix_, *Microbacterium*, *Hydrogenophaga*, and *Sphingopyxis* were only marginally detectable in the metagenome (<10 raw rDNA reads) of the two environmental samples A_3_ and B_mix_ (Figure 1) and not detected in the rRNA tag sequences of Marteinsson et al. (2013).

Remarkably, no evidence of members of Archaea in the lake was detected in the previous 16S rRNA amplicon-tag sequencing study (Marteinsson et al., 2013). This includes all known chemolithotrophic methanogens, which could potentially compete with homoacetogens for H_2_. In this metagenome sequencing-based study, which is not affected by PCR bias, we identified a significant number of archaea-like sequences in A_3_ (data not shown). Nevertheless, a detailed analysis of those reads shows that they are not randomly distributed over a complete reference archaeal 16S rDNA gene (Supplementary Figure 1) and are instead localised to two overlapping regions. The absence of flanking sequence to these clusters and their exclusive location indicates that those reads do not originate from a complete gene. Therefore, we are not able to detect the presence of archaea in the lake with our metagenomic approach. The DNA extraction method used might also be a bias with an incomplete lysis of recalcitrant archaeal cells as it was noted in previous subsurface communities (Webster et al., 2003).

Since light is absent in the lake, chemoautotrophy must be responsible for the microbial growth as primary producers such as in subglacial lakes Vostok or Whillans (Rogers et al., 2013; Vick-Majors et al., 2016). The metabolic pathways detected in a combination of A_3_ and B_mix_ for carbon fixation are shown in Figure 2. *Acetobacterium* sp. contains most of the genes involved in the Wood–Ljungdahl homoacetogenesis pathway. This pathway uses H_2_ as an electron donor and CO_2_ both as an electron acceptor and carbon source to generate acetyl-CoA. Acetogenesis seems to be an important energy source in this ecosystem and supports the previous findings that the lakes contain taxon with homoacetogens as the closest relatives (Gaidos et al., 2004, 2009; Marteinsson et al., 2013). The closest cultured relative to *Acetobacterium* is *Acetobacterium woodii*, a strain using the Wood–Ljungdahl (reverse acetyl-CoA) pathway and H_2_ to fix CO_2_ into acetate and maintain a sodium ion gradient for ATP synthesis (Poehlein et al., 2012). Prokaryotes living close to the thermodynamic limit, like methanogens and acetogens, use the reductive acetyl-coA pathway for both CO_2_ fixation and energy conservation (Ragsdale and Pierce, 2008; Thauer et al., 2008). We propose that environmental factors such as low temperature and high H_2_ concentration in this ecosystem favour acetogens over methanogens (Nozhevnikova et al., 1994). The absence of methanogens in this unique ecosystem could also potentially be explained by the presence of bacteria such as Sulphate Reducing Bacteria (SRB) that out-compete methanogens for hydrogen or produced acetate in the lake, with lower K_s_ values for H_2_ and acetate (Kristjansson et al., 1982; Schönheit et al., 1982).

Complete pathways for assimilatory sulphur reduction into sulphide were detected and mainly assigned to *Sulfurospirillum* sp. whereas the dissimilatory reduction of sulphur in sulphide was assigned mainly to *Acetobacterium* sp. (Figure 3A). This difference might be the result of *Sulfurospirillum* only performing assimilatory sulphate reduction (i.e., to produce organosulphur compounds) and *Acetobacterium* the dissimilatory one to conserve energy. *Sulfuricurvum* was described as a sulphur oxidiser (Han et al., 2012) and not as a sulphate reducer which can explain why assimilatory genes were mainly assigned to *Sulfurospirillum*. Moreover, the reason why *Sulfurospirillum* has *phsA* gene and not standard dissimilatory genes might be explained by the low sulphate concentration in the lake making sulphate reduction difficult. Sulphide production might be energetically unfavourable against the background of high sulphide in the lake (around 1 mM). Thus sulphur-cycling species might take sulphur only to sulphite and then cycle it back to sulphate or sulphur under microaerobic conditions. *Sulfuricurvum* sp. and *Sulfurospirillum* sp. also play a role in activating sulphur with a polysulphide reductase. These results emphasise the central role of sulphur species as sources of electrons (S^2–^, SO_4_^2–^, and S_2_O_3_^2–^) for chemolithoautotrophy in the lake and, as expected, in the metabolism of amino acids. Respiratory sulphate reduction is a common process in environments with a high sulphate concentration whereas in sulphate-depleted anoxic environments, acetogenesis is favoured over sulphate reduction (Laanbroek et al., 1982; Muyzer and Stams, 2008; Stams and Plugge, 2009).

No complete nitrogen metabolic pathways were found in the co-assembly metagenome except for nitrogen fixation. The genes involved in the nitrogen fixation pathway were detected and belong mainly to *Sulfuricurvum* sp. The genus *Sulfuricurvum* sp. has not been reported as being diazotrophic but its sequenced representative *Sulfuricurvum kujiense* YK-1^T^ also possesses the necessary genes (Kodama and Watanabe, 2004; Han et al., 2012). The dissimilatory nitrate reduction to ammonium, which is an important reaction of the reductive branch of the nitrogen cycle (Simon and Klotz, 2013), is mainly taxonomically affiliated with *Geobacter/Pedobacter* genus. Whereas nitrification is an important chemoautotrophic pathway of new organic carbon production in Subglacial Lake Whillans (Christner et al., 2014), none of the genes involved in this pathway were found in our metagenomes. The presence of *nir* (NADH-dependent) but absence of *nrf* (periplasmic) genes might imply that assimilatory reduction but not respiratory reduction (for energy conservation) might be occurring in this oligotrophic environment.

The study of microbial communities inhabiting subglacial lakes is of importance as such ecosystems remain underexplored. Only four subglacial lakes were sampled for microbial analyses at the time this paper was written (Gaidos et al., 2004, 2009; Christner et al., 2014; Priscu et al., 2021). The results in our research support the former hypothesis that was based on a taxonomy study, but it also gives insight into the metabolic pathways. The ecosystem in this subglacial lake appears to have a chemolithoautotrophic foundation with sulphur and carbon cycling that is fuelled by H_2_, CO_2_, and sulphur species originating from the geothermal activity and by O_2_ from melted ice. By identifying the potential metabolic pathways to specific taxa in the microbial assemblage, we have gained insight into the ecology of microbiomes in the water column of such an extreme environment. However, we should assume that other ecological niches may exist, e.g., on the bottom of the lake, in the lake sediments, or at the ice/lake interface, and these remain undiscovered. Moreover, nearly half of the sequence reads in our metagenomic library were not assigned and are a source for future advances. Also, new targeted sampling with larger volume is needed e.g., samples collected close to potential geothermal vents at the bottom of the lake might reveal thermophiles belonging to both the Bacteria and Archaea by sequencing and cultivation strategies. Even if analysed samples were collected in 2007, the results of this study might still correlate with the current situation of the lake as the latter has experienced similar conditions and has undergone continuous filling and draining (called jökulhlaup) since records have been kept.

Finally, due to recent Arctic warming, ice caps are thinning, notably in Iceland (Gudmundsson et al., 2008). Despite their isolation from the surface, these lakes are influenced by the dynamics and melting of glaciers, which are changing with climate change (Livingstone et al., 2022). The existence of subglacial lakes is thus threatened in the long run. The complete exploration of the microbial diversity of this ecosystem needs to happen quickly to monitor such future changes.

## Data availability statement

The data presented in this study are deposited in NCBI Sequences Read Archive repository under the accession number: SRP011365 (https://www.ncbi.nlm.nih.gov/sra/?term=SRP011365).

## Author contributions

PV, GF, and VM conceived and designed the study and analysed the data. AK performed the DNA extractions. GF performed the bioinformatic analyses. PV, GF, EG, and VM wrote the main manuscript text. All authors reviewed the manuscript.

## Footnotes

1
https://github.com/gfarrant/astrolakes

## References

[B1] Achberger A. M. Christner B. C. Michaud A. B. Priscu J. C. Skidmore M. L. Vick-Majors T. J. (2016). Microbial community structure of subglacial lake whillans. West Antarctica. *Front. Microbiol.* 7:1457. 10.3389/fmicb.2016.01457 27713727 PMC5032586

[B2] Agustsdottir A. M. Brantley S. L. (1994). Volatile fluxes integrated over 4 decades at Grímsvötn volcano, Iceland. *J. Geophys. Research-Solid Earth* 99 9505–9522. 10.1029/93JB03597

[B3] Alneberg J. Bjarnason B. S. De Bruijn I. Schirmer M. Quick J. Ijaz U. Z. (2014). Binning metagenomic contigs by coverage and composition. *Nat. Methods* 11 1144–1146. 10.1038/nmeth.3103 25218180

[B4] Balch W. E. Fox G. E. Magrum L. J. Woese C. R. Wolfe R. S. (1979). Methanogens: re-evaluation of a unique biological group. *Microbiol. Rev.* 43 260–296. 10.1128/mr.43.2.260-296.1979 390357 PMC281474

[B5] Bankevich A. Nurk S. Antipov D. Gurevich A. A. Dvorkin M. Kulikov A. S. (2012). SPAdes: a new genome assembly algorithm and its applications to single-cell sequencing. *J. Comp. Biol.* 19 455–477. 10.1089/cmb.2012.0021 22506599 PMC3342519

[B6] Bjornsson H. (2003). Subglacial lakes and jokulhlaups in Iceland. *Global Plan. Change* 35 255–271. 10.1016/S0921-8181(02)00130-3

[B7] Brown C. T. Hug L. A. Thomas B. C. Sharon I. Castelle C. J. Singh A. (2015). Unusual biology across a group comprising more than 15% of domain Bacteria. *Nature* 523:208. 10.1038/nature14486 26083755

[B8] Brumfield K. D. Huq A. Colwell R. R. Olds J. L. Leddy M. B. (2020). Microbial resolution of whole genome shotgun and 16S amplicon metagenomic sequencing using publicly available NEON data. *PLoS One* 15:e0228899. 10.1371/journal.pone.0228899 32053657 PMC7018008

[B9] Buchfink B. Xie C. Huson D. H. (2015). Fast and sensitive protein alignment using DIAMOND. *Nat. Methods* 12:59. 10.1038/nmeth.3176 25402007

[B10] Bulat S. A. (2016). Microbiology of the subglacial Lake Vostok: first results of borehole-frozen lake water analysis and prospects for searching for lake inhabitants. *Philos. Trans. R. Soc. Mathematical Phys. Eng. Sci.* 374:20140292. 10.1098/rsta.2014.0292 26667905

[B11] Camacho C. Coulouris G. Avagyan V. Ma N. Papadopoulos J. Bealer K. (2009). BLAST+: architecture and applications. *BMC Bioinformatics* 10:421. 10.1186/1471-2105-10-421 20003500 PMC2803857

[B12] Chinn T. (1993). Physical hydrology of the dry valley lakes. *Phys. Biogeochem. Proc. Antarctic Lakes* 59 1–51. 10.1029/AR059p0001

[B13] Christner B. C. Mosley-Thompson E. Thompson L. G. Reeve J. N. (2001). Isolation of bacteria and 16S rDNAs from Lake Vostok accretion ice. *Environ. Microbiol.* 3 570–577. 10.1046/j.1462-2920.2001.00226.x 11683867

[B14] Christner B. C. Priscu J. C. Achberger A. M. Barbante C. Carter S. P. Christianson K. (2014). A microbial ecosystem beneath the West Antarctic ice sheet. *Nature* 512 310–313. 10.1038/nature13667 25143114

[B15] Dartnell L. R. Hunter S. J. Lovell K. V. Coates A. J. Ward J. M. (2010). Low-temperature ionizing radiation resistance of *Deinococcus radiodurans* and Antarctic Dry Valley bacteria. *Astrobiology* 10 717–732. 10.1089/ast.2009.0439 20950171

[B16] Davies B. J. Hambrey M. J. Glasser N. F. Holt T. Rodés A. Smellie J. L. (2017). Ice-dammed lateral lake and epishelf lake insights into Holocene dynamics of marguerite trough ice stream and george VI Ice Shelf, Alexander Island, Antarctic Peninsula. *Quaternary Sci. Rev.* 177 189–219. 10.1016/j.quascirev.2017.10.016

[B17] Degryse E. Glansdorff N. Pierard A. (1978). Comparative analysis of extreme thermophilic bacteria belonging to genus Thermus. *Arch. Microbiol.* 117 189–196. 10.1007/BF00402307 678024

[B18] Eisenmann E. Beuerle J. Sulger K. Kroneck P. M. Schumacher W. (1995). Lithotrophic growth of *Sulfurospirillum deleyianum* with sulfide as electron donor coupled to respiratory reduction of nitrate to ammonia. *Arch. Microbiol.* 164 180–185. 10.1007/BF02529969

[B19] Ferrari B. Winsley T. Ji M. Neilan B. (2014). Insights into the distribution and abundance of the ubiquitous candidatus Saccharibacteria phylum following tag pyrosequencing. *Sci. Rep.* 4:3957. 10.1038/srep03957 24492458 PMC5379237

[B20] Gaidos E. Lanoil B. Thorsteinsson T. Graham A. Skidmore M. Han S. K. (2004). A viable microbial community in a subglacial volcanic crater lake. Iceland. *Astrobiology* 4 327–344. 10.1089/ast.2004.4.327 15383238

[B21] Gaidos E. Marteinsson V. Thorsteinsson T. Johannesson T. Runarsson A. R. Stefansson A. (2009). An oligarchic microbial assemblage in the anoxic bottom waters of a volcanic subglacial lake. *ISME J.* 3 486–497. 10.1038/ismej.2008.124 19092861

[B22] González-Toril E. Amils R. Delmas R. J. Petit J.-R. Komárek J. Elster J. (2009). Bacterial diversity of autotrophic enriched cultures from remote, glacial Antarctic, Alpine and Andean aerosol, snow and soil samples. *Biogeosciences* 6 33–44. 10.5194/bg-6-33-2009

[B23] Gudmundsson M. T. Larsen G. Höskuldsson Á Gylfason ÁG. (2008). Volcanic hazards in Iceland. *Jokull* 58 251–268. 10.33799/jokull2008.58.251

[B24] Gura C. Rogers S. O. (2020). Metatranscriptomic and metagenomic analysis of biological diversity in subglacial lake vostok (Antarctica). *Biology* 9:55. 10.3390/biology9030055 32188079 PMC7150893

[B25] Han C. Kotsyurbenko O. Chertkov O. Held B. Lapidus A. Nolan M. (2012). Complete genome sequence of the sulfur compounds oxidizing chemolithoautotroph *Sulfuricurvum kujiense* type strain (YK-1 T). *Standards Genomic Sci.* 6:94. 10.4056/sigs.2456004 22675602 PMC3368400

[B26] Howard-Williams C. Schwarz A.-M. Hawes I. Priscu J. (1998). Optical properties of the McMurdo Dry Valley lakes. *Antarctic Res. Ser.* 72 189–203. 10.1029/AR072p0189

[B27] Huang Y. Gilna P. Li W. (2009). Identification of ribosomal RNA genes in metagenomic fragments. *Bioinformatics* 25 1338–1340. 10.1093/bioinformatics/btp161 19346323 PMC2677747

[B28] Johannesson T. Thorsteinsson T. Stefansson A. Gaidos E. J. Einarsson B. (2007). Circulation and thermodynamics in a subglacial geothermal lake under the Western Skafta cauldron of the Vatnajokull ice cap, Iceland. *Geophys. Res. Lett.* 34 502–508. 10.1029/2007GL030686

[B29] Kanehisa M. Goto S. (2000). KEGG: kyoto encyclopedia of genes and genomes. *Nucleic Acids Res.* 28 27–30. 10.1093/nar/28.1.27 10592173 PMC102409

[B30] Kanehisa M. Sato Y. Kawashima M. Furumichi M. Tanabe M. (2015). KEGG as a reference resource for gene and protein annotation. *Nucleic Acids Res.* 44 D457–D462. 10.1093/nar/gkv1070 26476454 PMC4702792

[B31] Karl D. Bird D. Björkman K. Houlihan T. Shackelford R. Tupas L. (1999). Microorganisms in the accreted ice of Lake Vostok, Antarctica. *Science* 286 2144–2147. 10.1126/science.286.5447.2144 10591643

[B32] Kirschvink J. L. Gaidos E. J. Bertani L. E. Beukes N. J. Gutzmer J. Maepa L. N. (2000). Paleoproterozoic snowball Earth: extreme climatic and geochemical global change and its biological consequences. *Proc. Natl. Acad. Sci. U S A.* 97 1400–1405. 10.1073/pnas.97.4.1400 10677473 PMC26445

[B33] Kivelson M. G. Khurana K. K. Russell C. T. Volwerk M. Walker R. J. Zimmer C. (2000). Galileo magnetometer measurements: a stronger case for a subsurface ocean at Europa. *Science* 289 1340–1343. 10.1126/science.289.5483.1340 10958778

[B34] Kivelson M. G. Khurana K. K. Volwerk M. (2002). The permanent and inductive magnetic moments of ganymede. *Icarus* 157 507–522. 10.1006/icar.2002.6834

[B35] Kodama Y. Watanabe K. (2004). *Sulfuricurvum kujiense* gen. nov., sp nov., a facultatively anaerobic, chemolithoautotrophic, sulfur-oxidizing bacterium isolated from an underground crude-oil storage cavity. *Int. J. Systematic Evol. Microbiol.* 54 2297–2300. 10.1099/ijs.0.63243-0 15545474

[B36] Kristjansson J. K. Schönheit P. Thauer R. K. (1982). Different K s values for hydrogen of methanogenic bacteria and sulfate reducing bacteria: an explanation for the apparent inhibition of methanogenesis by sulfate. *Arch. Microbiol.* 131 278–282. 10.1007/BF00405893

[B37] Laanbroek H. J. Abee T. Voogd I. L. (1982). Alcohol conversion by *Desulfobulbus propionicus* Lindhorst in the presence and absence of sulfate and hydrogen. *Arch. Microbiol.* 133 178–184. 10.1007/BF00414998

[B38] Lauro S. E. Pettinelli E. Caprarelli G. Guallini L. Rossi A. P. Mattei E. (2020). Multiple subglacial water bodies below the south pole of Mars unveiled by new MARSIS data. *Nat. Astronomy* 5 63–70. 10.1038/s41550-020-1200-6

[B39] Livingstone S. J. Li Y. Rutishauser A. Sanderson R. J. Winter K. Mikucki J. A. (2022). Subglacial lakes and their changing role in a warming climate. *Nat. Rev. Earth Environ.* 3 106–124. 10.1038/s43017-021-00246-9

[B40] Lloyd K. G. Steen A. D. Ladau J. Yin J. Crosby L. (2018). Phylogenetically novel uncultured microbial cells dominate earth microbiomes. *mSystems* 3:e00055-18. 10.1128/mSystems.00055-18 30273414 PMC6156271

[B41] Logares R. Sunagawa S. Salazar G. Cornejo-Castillo F. M. Ferrera I. Sarmento H. (2014). Metagenomic 16S rDNA I llumina tags are a powerful alternative to amplicon sequencing to explore diversity and structure of microbial communities. *Environ. Microbiol.* 16 2659–2671. 10.1111/1462-2920.12250 24102695

[B42] Magoč T. Salzberg S. L. (2011). FLASH: fast length adjustment of short reads to improve genome assemblies. *Bioinformatics* 27 2957–2963. 10.1093/bioinformatics/btr507 21903629 PMC3198573

[B43] Marteinsson V. T. Birrien J. L. Prieur D. (1997). In situ enrichment and isolation of thermophilic microorganisms from deep-sea vent environments. *Canadian J. Microbiol.* 43 694–697. 10.1139/m97-100

[B44] Marteinsson V. T. Kristjansson J. K. Kristmannsdottir H. Dahlkvist M. Saemundsson K. Hannington M. (2001). Discovery and description of giant submarine smectite cones on the seafloor in Eyjafjordur, northern Iceland, and a novel thermal microbial habitat. *Appl. Environ. Microbiol.* 67 827–833. 10.1128/AEM.67.2.827-833.2001 11157250 PMC92654

[B45] Marteinsson V. T. Runarsson A. Stefansson A. Thorsteinsson T. Johannesson T. Magnusson S. H. (2013). Microbial communities in the subglacial waters of the Vatnajokull ice cap. Iceland. *ISME J.* 7 427–437. 10.1038/ismej.2012.97 22975882 PMC3554413

[B46] Mori K. Yamaguchi K. Sakiyama Y. Urabe T. Suzuki K.-I. (2009). Caldisericum exile gen. nov., sp. nov., an anaerobic, thermophilic, filamentous bacterium of a novel bacterial phylum, *Caldiserica phyl*. nov., originally called the candidate phylum OP5, and description of *Caldisericaceae fam*. nov., *Caldisericales ord*. nov. and *Caldisericia classis* nov. *Int. J. Systematic Evol. Microbiol.* 59 2894–2898. 10.1099/ijs.0.010033-0 19628600

[B47] Muyzer G. Stams A. J. M. (2008). The ecology and biotechnology of sulphate-reducing bacteria. *Nat. Rev. Microbiol.* 6 441–454. 10.1038/nrmicro1892 18461075

[B48] Nozhevnikova A. N. Kotsyurbenko O. R. Simankova M. V. (1994). “Acetogenesis at low temperature,” in *Acetogenesis. Chapman & Hall Microbiology Series*, ed. Drake H. L. (Boston, MA: Springer), 416–431. 10.1007/978-1-4615-1777-1_15

[B49] Palmer S. J. Dowdeswell J. A. Christoffersen P. Young D. A. Blankenship D. D. Greenbaum J. S. (2013). Greenland subglacial lakes detected by radar. *Geophys. Res. Lett.* 40 6154–6159. 10.1002/2013GL058383 26450175

[B50] Parada A. E. Needham D. M. Fuhrman J. A. (2016). Every base matters: assessing small subunit rRNA primers for marine microbiomes with mock communities, time series and global field samples. *Environ. Microbiol.* 18 1403–1414. 10.1111/1462-2920.13023 26271760

[B51] Peng Y. Leung H. C. Yiu S.-M. Chin F. Y. (2012). IDBA-UD: a de novo assembler for single-cell and metagenomic sequencing data with highly uneven depth. *Bioinformatics* 28 1420–1428. 10.1093/bioinformatics/bts174 22495754

[B52] Peter H. Sommaruga R. (2016). Shifts in diversity and function of lake bacterial communities upon glacier retreat. *ISME J.* 10:1545. 10.1038/ismej.2015.245 26771929 PMC4852812

[B53] Poehlein A. Schmidt S. Kaster A.-K. Goenrich M. Vollmers J. Thürmer A. (2012). An ancient pathway combining carbon dioxide fixation with the generation and utilization of a sodium ion gradient for ATP synthesis. *PLoS One* 7:e33439. 10.1371/journal.pone.0033439 22479398 PMC3315566

[B54] Priscu J. C. Adams E. E. Lyons W. B. Voytek M. A. Mogk D. W. Brown R. L. (1999). Geomicrobiology of subglacial ice above Lake Vostok. Antarctica. *Science* 286 2141–2144. 10.1126/science.286.5447.2141 10591642

[B55] Priscu J. C. Kalin J. Winans J. Campbell T. Siegfried M. R. Skidmore M. (2021). Scientific access into Mercer Subglacial Lake: scientific objectives, drilling operations and initial observations. *Ann. Glaciol.* 62 340–352. 10.1017/aog.2021.10

[B56] Quast C. Pruesse E. Yilmaz P. Gerken J. Schweer T. Yarza P. (2012). The SILVA ribosomal RNA gene database project: improved data processing and web-based tools. *Nucleic Acids Res.* 41 D590–D596. 10.1093/nar/gks1219 23193283 PMC3531112

[B57] Ragsdale S. W. Pierce E. (2008). Acetogenesis and the Wood–Ljungdahl pathway of CO2 fixation. *Biochim. Biophys. Acta (BBA)-Proteins Proteom.* 1784 1873–1898. 10.1016/j.bbapap.2008.08.012 18801467 PMC2646786

[B58] Reasoner D. J. Geldreich E. E. (1985). A new medium for the enumeration and subculture of bacteria from potable water. *Appl. Environ. Microbiol.* 49 1–7. 10.1128/aem.49.1.1-7.1985 3883894 PMC238333

[B59] Rogers S. Shtarkman Y. Koçer Z. Edgar R. Veerapaneni R. Elia T. (2013). Ecology of subglacial lake vostok (Antarctica), based on metagenomic/metatranscriptomic analyses of accretion ice. *Biology* 2 629. 10.3390/biology2020629 24832801 PMC3960894

[B60] Rutishauser A. Blankenship D. D. Sharp M. Skidmore M. L. Greenbaum J. S. Grima C. (2018). Discovery of a hypersaline subglacial lake complex beneath Devon Ice Cap, Canadian Arctic. *Sci. Adv.* 4:eaar4353. 10.1126/sciadv.aar4353 29651462 PMC5895444

[B61] Schönheit P. Kristjansson J. K. Thauer R. K. (1982). Kinetic mechanism for the ability of sulfate reducers to out-compete methanogens for acetate. *Arch. Microbiol.* 132 285–288. 10.1007/BF00407967

[B62] Simon J. Klotz M. G. (2013). Diversity and evolution of bioenergetic systems involved in microbial nitrogen compound transformations. *Biochim. Biophys. Acta (BBA)-Bioenergetics* 1827 114–135. 10.1016/j.bbabio.2012.07.005 22842521

[B63] Stams A. J. Plugge C. M. (2009). Electron transfer in syntrophic communities of anaerobic bacteria and archaea. *Nat. Rev. Microbiol.* 7 568–577. 10.1038/nrmicro2166 19609258

[B64] Stoddard S. F. Smith B. J. Hein R. Roller B. R. Schmidt T. M. (2014). rrnDB: improved tools for interpreting rRNA gene abundance in bacteria and archaea and a new foundation for future development. *Nucleic Acids Res.* 43 D593–D598. 10.1093/nar/gku1201 25414355 PMC4383981

[B65] Stolz J. F. Ellis D. J. Blum J. S. Ahmann D. Lovley D. R. Oremland R. S. (1999). *Sulfurospirillum barnesii* sp nov and *Sulfurospirillum arsenophilum* sp nov., new members of the *Sulfurospirillum clade* of the epsilon Proteobacteria. *Int. J. Systematic Bacteriol.* 49 1177–1180. 10.1099/00207713-49-3-1177 10425777

[B66] Thauer R. K. Kaster A.-K. Seedorf H. Buckel W. Hedderich R. (2008). Methanogenic archaea: ecologically relevant differences in energy conservation. *Nat. Rev. Microbiol.* 6 579–591. 10.1038/nrmicro1931 18587410

[B67] Thomas P. C. Tajeddine R. Tiscareno M. S. Burns J. A. Joseph J. Loredo T. J. (2016). Enceladus’s measured physical libration requires a global subsurface ocean. *Icarus* 264 37–47. 10.1016/j.icarus.2015.08.037

[B68] Thorsteinsson T. Elefsen S. O. Gaidos E. Lanoil B. Johannesson T. Kjartansson V. (2008). A hot water drill with built-in sterilization: design, testing and performance. *Jokull* 51 71–82. 10.33799/jokull2007.57.071o

[B69] Trivedi C. B. Lau G. E. Grasby S. E. Templeton A. S. Spear J. R. (2018). Low-temperature sulfidic-ice microbial communities, borup fiord pass, Canadian high Arctic. *Front. Microbiol.* 9:1622. 10.3389/fmicb.2018.01622 30087659 PMC6066561

[B70] Tulaczyk S. Mikucki J. A. Siegfried M. R. Priscu J. C. Barcheck C. G. Beem L. H. (2014). WISSARD at Subglacial Lake Whillans, West Antarctica: scientific operations and initial observations. *Ann. Glaciol.* 55 51–58. 10.3189/2014AoG65A009

[B71] Vick-Majors T. J. Mitchell A. C. Achberger A. M. Christner B. C. Dore J. E. Michaud A. B. (2016). Physiological ecology of microorganisms in subglacial lake whillans. *Front. Microbiol.* 7:1705. 10.3389/fmicb.2016.01705 27833599 PMC5081474

[B72] Wang Q. Garrity G. M. Tiedje J. M. Cole J. R. (2007). Naive Bayesian classifier for rapid assignment of rRNA sequences into the new bacterial taxonomy. *Appl. Environ. Microbiol.* 73 5261–5267. 10.1128/AEM.00062-07 17586664 PMC1950982

[B73] Webster G. Newberry C. J. Fry J. C. Weightman A. J. (2003). Assessment of bacterial community structure in the deep sub-seafloor biosphere by 16S rDNA-based techniques: a cautionary tale. *J. Microbiol. Methods* 55 155–164. 10.1016/S0167-7012(03)00140-4 14500007

[B74] Yilmaz P. Parfrey L. W. Yarza P. Gerken J. Pruesse E. Quast C. (2013). The SILVA and “All-species Living Tree Project (LTP)” taxonomic frameworks. *Nucleic Acids Res*. 42, D643–D648. 10.1093/nar/gkt1209 24293649 PMC3965112

[B75] Zhou Z. Jiang F. Wang S. Peng F. Dai J. Li W. (2012). *Pedobacter arcticus* sp. nov., a facultative psychrophile isolated from Arctic soil, and emended descriptions of the genus *Pedobacter*, *Pedobacter heparinus*, *Pedobacter daechungensis*, *Pedobacter terricola*, *Pedobacter glucosidilyticus* and *Pedobacter lentus*. *Int. J. Systematic Evol. Microbiol.* 62 1963–1969. 10.1099/ijs.0.031104-0 22003034

[B76] Zhu W. Lomsadze A. Borodovsky M. (2010). Ab initio gene identification in metagenomic sequences. *Nucleic Acids Res.* 38:e132. 10.1093/nar/gkq275 20403810 PMC2896542

