# Supplementary figures and images for: Metagenomic analyses of a microbial assemblage in a subglacial lake beneath the Vatnajökull ice cap, Iceland
Source: Front Microbiol. 2023 Mar 30;14:1122184. doi: 10.3389/fmicb.2023.1122184 (PMC10098204; doi:10.3389/fmicb.2023.1122184)

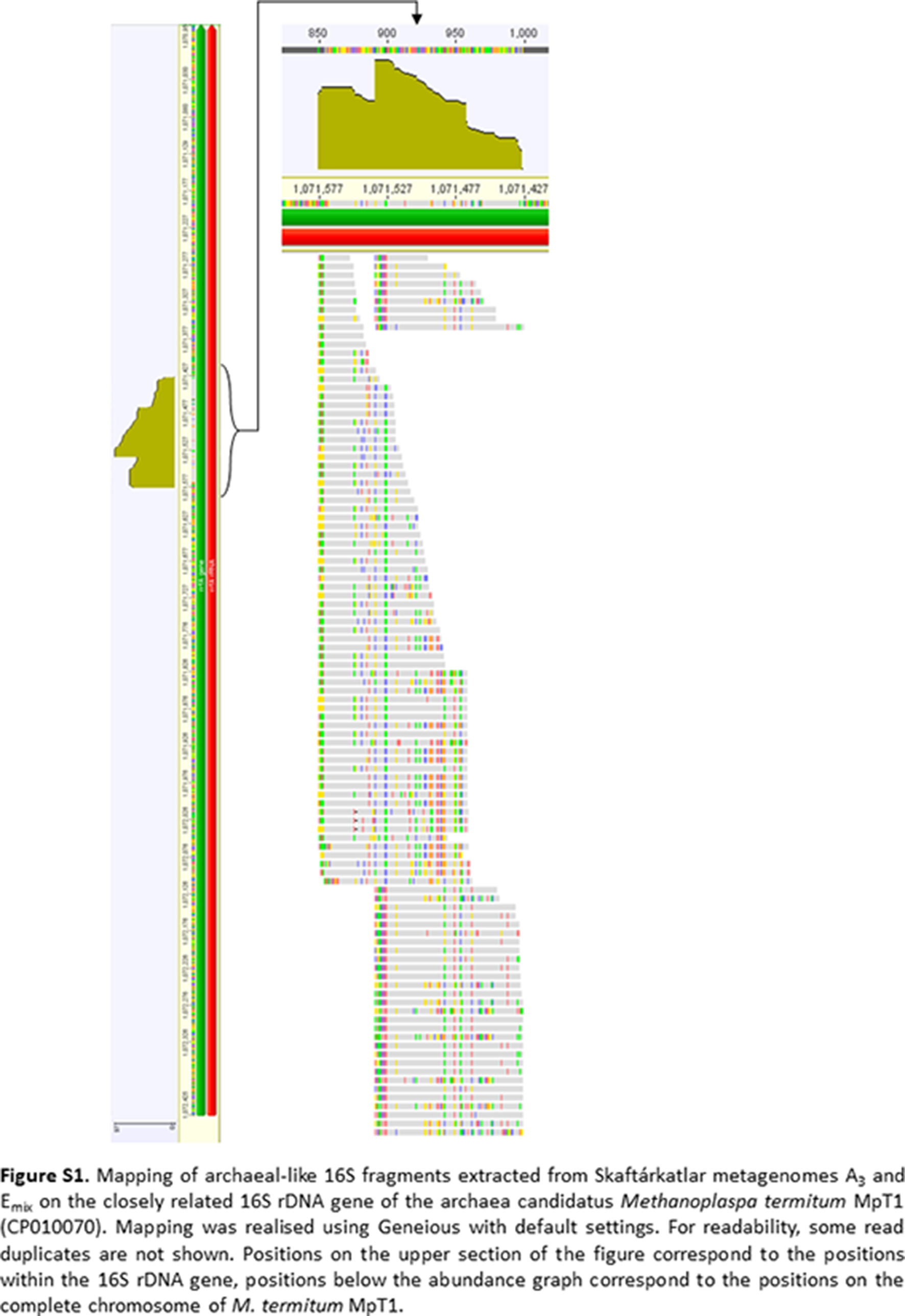

Supplement: Supplementary file 1 [file Image_1.TIF]
