# Supplementary material for: Metagenomic analyses of a microbial assemblage in a subglacial lake beneath the Vatnajökull ice cap, Iceland
Source: Front Microbiol. 2023 Mar 30;14:1122184. doi: 10.3389/fmicb.2023.1122184 (PMC10098204; doi:10.3389/fmicb.2023.1122184)
Supplement: Supplementary file 2 [file Table_1.DOCX]

**Table S1**: Chemistry of the samples from Lake East Skaftárkatlar

| **Sample** | **units** | **A3** | **B1** | **B2** | **B3** | **B4** |
| --- | --- | --- | --- | --- | --- | --- |
| *Depth* | *meters* | *379* | *284* | *336* | *377* | *388* |
| ΣCO_2_ |  | 122.49 | 106.28 | 121.47 | 96.16 | 112.89 |
| SiO_2_ | ppm | 101.74 | 99.61 | 98.32 | 97.94 | 99.23 |
| Na | ppm | 30.65 | 30.04 | 29.75 | 29.60 | 30.44 |
| K | ppm | 3.460 | 3.390 | 3.350 | 3.350 | 3.240 |
| Ca | ppm | 3.490 | 3.461 | 3.423 | 3.452 | 4.741 |
| Mg | ppm | 0.250 | 0.245 | 0.247 | 0.239 | 0.305 |
| Fe | ppm | 0.262 | 0.161 | 0.235 | 0.179 | 0.332 |
| Al | ppm | 0.093 | 0.083 | 0.102 | 0.077 | 0.086 |
| Sr | ppm | 0.0063 | 0.0049 | 0.0049 | 0.0053 | 0.0089 |
| B | ppm | 0.520 | 0.518 | 0.513 | 0.510 | 0.525 |
| F | ppm | 0.1218 | 0.1069 | 0.1063 | 0.106 | 0.1144 |
| Cl | ppm | 12.2754 | 10.1552 | 10.1639 | 9.9656 | 10.6635 |
| SO_4_ | ppm | **4.71** | 0.29 | 1.08 | 1.03 | 1.43 |
| H_2_S | ppm | 11.54 | 14.36 | 10.30 | 15.04 | 25.50 |
| NO_2_ | ppb | **156** | 66 | 3.0832 | 4 | 54 |
| NO_3_ | ppb | 8.3 | 1.2 | 4.0832 | n.d. | 30.02 |
| PO_4_ | ppb | 1.4 | n.d. | 5.0832 | n.d. | n.d. |
