# Supplementary material for: Metagenomic analyses of a microbial assemblage in a subglacial lake beneath the Vatnajökull ice cap, Iceland
Source: Front Microbiol. 2023 Mar 30;14:1122184. doi: 10.3389/fmicb.2023.1122184 (PMC10098204; doi:10.3389/fmicb.2023.1122184)
Supplement: Supplementary file 3 [file Table_2.DOCX]

**Table S2.** Whole gene recruitment of the Skafárkatlar metagenomes on reference genomes

|  | **Strain Name** | **Total number  of Genes** | **Number of genes with 1+ reads coverage** | **Ratio** | **Number of genes with 10+ reads coverage** | **Ratio** |
| --- | --- | --- | --- | --- | --- | --- |
| identity ≥ 80 % | *Sulfuricurvum kujiense* | 2,853 | 2,178 | 76.3 % | 1,882 | 66.0 % |
|  | *Sulfurospirillum halorespirans* | 3,033 | 2,541 | 83.8 % | 2,270 | 74.8 % |
|  | *Acetobacterium woodii* | 3,654 | 2,344 | 64.1 % | 1,862 | 51.0 % |
|  | *Pelobacter propionicus* | 3,901 | 2,244 | 57.5 % | 1,760 | 45.1 % |
|  | *Candidatus Saccharibacteria* | 1,874 | 803 | 42.8 % | 583 | 31.1 % |
|  | *Caldisericum exile* | 1,568 | 678 | 43.2 % | 235 | 15.0 % |
|  | *Prolixibacter denitrificans* | 4,048 | 1,871 | 46.2 % | 1,172 | 29.0 % |
| identity ≥ 90 % | *Sulfuricurvum kujiense* | 2,853 | 1,008 | 35.3 % | 556 | 19.5 % |
|  | *Sulfurospirillum halorespirans* | 3,033 | 1,548 | 51.0 % | 758 | 25.0 % |
|  | *Acetobacterium woodii* | 3,654 | 1,013 | 27.7 % | 472 | 12.9 % |
|  | *Pelobacter propionicus* | 3,901 | 950 | 24.4 % | 389 | 10.0 % |
|  | *Candidatus Saccharibacteria* | 1,874 | 271 | 14.5 % | 119 | 6.4 % |
|  | *Caldisericum exile* | 1,568 | 185 | 11.8 % | 56 | 3.6 % |
|  | *Prolixibacter denitrificans* | 4,048 | 544 | 13.4 % | 128 | 3.2 % |
